# Long noncoding RNA ArfGAP with RhoGAP domain, ankyrin repeat and PH domain 1 antisense RNA 1 recruits enhancer of zeste 2 polycomb repressive complex 2 subunit to promote the proliferation, migration and invasion of lung adenocarcinoma cells

**DOI:** 10.1080/21655979.2022.2050968

**Published:** 2022-03-15

**Authors:** Jinyuan Liu, Chunfeng Pan, Rongxin Lu, Shijiang Zhang

**Affiliations:** Department of Thoracic and Cardiovascular Surgery, The First Affiliated Hospital of Nanjing Medical University, Nanjing, Jiangsu, China

**Keywords:** Lung adenocarcinoma, ARAP1-AS1, ARAP1, EZH2

## Abstract

The detailed function of ARAP1-AS1, the antisense RNA of Arf-GAP with Rho-GAP domain, ANK repeat and PH domain-containing protein 1 (ARAP1), in lung adenocarcinoma (LUAD) has not been clearly elucidated and required further investigation. Our study is committed to exploring the role of ARAP1-AS1 in LUAD. Gene expression in LUAD was measured by real-time quantitative polymerase-chain reaction (RT-qPCR). The influence of ARAP1-AS1 on LUAD cell malignant behaviors was evaluated by 3-(4,5-Dimethylthiazol-2-yl)-2,5-diphenyltetrazolium bromide (MTT) assay, colony formation assay, Transwell invasion assay and wound healing assay. Subcellular fractionation assay detected the cellular localization of ARAP1-AS1 in LUAD. The protein levels were subjected to western blotting. RNA immunoprecipitation (RIP) and luciferase reporter assay were employed to verify the interaction between ARAP1-AS1, ARAP1 and enhancer of zeste 2 polycomb repressive complex 2 subunit (EZH2). Our investigation identified that ARAP1-AS1 was upregulated in LUAD cells and tissues. ARAP1-AS1 silencing repressed LUAD cell growth and migration. Furthermore, ARAP1-AS1 knockdown altered the expression of its sense mRNA, ARAP1. ARAP1-AS1 could recruit EZH2 to inhibit ARAP1 expression. Additionally, the downregulation of ARAP1 reversed ARAP1-AS1 downregulation-induced repression of cell growth and migration in LUAD. In conclusion, ARAP1-AS1 recruited EZH2 to silence ARAP1, facilitating cell proliferation, migration and invasion in LUAD. Our study demonstrated the possibility of ARAP1-AS1 to be a novel therapeutic target for LUAD.

**Figure:**
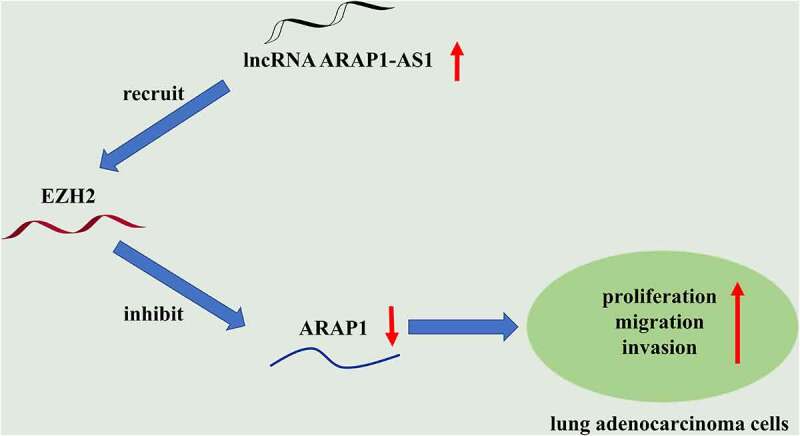

## 1. Introduction

Lung cancer is one of the most common cancers and the leading cause of cancer-related death worldwide [1]. Every year, approximately 1.6 million people are diagnosed with lung cancer [2]. Lung adenocarcinoma (LUAD) accounts for about 40% of all lung cancer cases [3]. LUAD has no obvious special symptoms in the early stage, and deteriorates rapidly in the late stage, requiring timely symptomatic treatment [4,5]. The treatment of LUAD includes thoracic surgery for early stage LUAD, radiotherapy such as stereotactic ablative radiotherapy (SABR), and percutaneous radiofrequency ablation (RFA) for medically inoperable patients [6]. Despite major advances in early diagnosis and treatment of LUAD, most LUAD patients die of recurrence and metastasis due to frequent hematogenous metastasis [7]. Therefore, the molecular mechanisms associated with the tumorigenesis and metastasis of LUAD should be identified to help understand the pathological progress of LUAD and then design targeted therapeutic drugs [8].

Long noncoding RNAs (lncRNAs) are RNAs over 200 nucleotides in length with very low levels of expression and sequence conservation [9]. LncRNAs regulate multiple biological functions at the transcriptional and post-transcriptional levels, or directly modulate protein activity [10]. Increasing studies have demonstrated that various differentially expressed lncRNAs are potential biological regulators in many cellular activities such as cell differentiation, growth and metastasis [11–13]. Multiple lncRNAs were reported to regulate cell proliferation and metastasis during the tumorigenesis of LUAD. For example, lncRNA small nucleolar RNA host gene 14 (SNHG14) is upregulated in LUAD, and SNHG14 enhances LUAD cell proliferation and invasion by targeting microRNA-613 [14]. LncRNA titin antisense RNA 1 (TTN-AS1) promotes cell proliferation, migration, invasion, and epithelial-mesenchymal transition in LUAD by binding with microRNA-4677-3p and increasing the expression of zinc finger E-box binding homeobox 1 (ZEB1) [15]. LncRNA ARAP1-AS1, the antisense RNA of Arf-GAP with Rho-GAP domain, ANK repeat and PH domain-containing protein 1 (ARAP1), was also reported to participate in the development of many human cancers and the underlying molecular mechanisms have been identified. For example, ARAP1-AS1 exhibits a high level in bladder cancer cells, and ARAP1-AS1 enhances tumor cell proliferation, migration and invasion via regulating the microRNA-4735-3p/notch receptor 2 axis [16]. ARAP1-AS1 promotes cell proliferation and migration in clear cell renal cell carcinoma by regulating the microRNA-361-3p/placental growth factor axis [17]. Previously, ARAP1-AS1 was reported to be upregulated in lung cancer and ARAP1-AS1 knockdown significantly suppresses the proliferation of lung cancer cells and induce G0/G1 cell cycle arrest by reducing the expression of cell cycle-related protein cyclin D1 [18]. However, whether ARAP1-AS1modulates LUAD cell migration and invasion was not investigated before.

In our study, we aimed to explore the detailed function and regulatory mechanism of ARAP1-AS1 in the malignant behaviors of LUAD cells. We hypothesized that ARAP1-AS1 facilitated LUAD cell proliferation, migration and invasion via recruiting enhancer of zeste 2 polycomb repressive complex 2 subunit (EZH2) to silence ARAP1. Our finding might provide a novel therapeutic target for LUAD treatment.

## 2. Materials and methods

### 2.1. Bioinformatics analysis

The expression of ARAP1-AS1 and ARAP1 in normal tissues (n = 347) and LUAD tissues (n = 483) was predicted in GEPIA database (http://gepia2.cancer-pku.cn/) [19]. EZH2 expression in normal tissues (n = 59) and LUAD tissues (n = 526) was detected by ENCORI database (http://starbase.sysu.edu.cn/) [20]. The correlation between ARAP1 and overall survival time of patients diagnosed with LUAD was predicted according to Kaplan-Meier Plotter website (https://kmplot.com/) [21].

### 2.2. Cell culture

Two LUAD cell lines (H1975 and A549) and a normal human bronchial epithelial cell line BEAS-2B were obtained from American Type Culture Collection (ATCC; Manassas, VA, USA). All cells were incubated in RPMI-1640 medium (Hyclone, USA) containing 10% fetal bovine serum (FBS; Gibco, USA) and maintained at 37°C in a humidified atmosphere with 5% CO_2_ [22].

### 2.3. RNA interference

Two ARAP1-AS1 shRNAs (sh-ARAP1-AS1 #1, and #2), two EZH2 shRNAs (sh-EZH2 #1, and #2), ARAP1 shRNA, and scrambled negative control shRNA (sh-NC) were bought from GenePharma (Shanghai, China). LUAD cells were seeded in 6-well plates, and were transfected with the above plasmids using Lipofectamine 2000 (Invitrogen, Carlsbad, CA, USA) 24 h later [23]. A549 and H1975 cells were harvested for real-time quantitative polymerase-chain reaction (RT-qPCR) 48 h post transfection.

### 2.4. RNA isolation and RT-qPCR

Total RNA was extracted from LUAD cells or tissues using TRIzol reagent (Invitrogen). A PrimeScript RT Kit (Takara, Dalian, China) was used to reverse transcribe total RNA into complementary DNA. RT-qPCR was performed on Eco Real-Time PCR System (Illumina Inc., San Diego, CA, USA) using SYBR Premix Ex Taq (Takara). Glyceraldehyde-3-phosphate dehydrogenase (GAPDH) served as the internal control. Gene expression was quantified using the 2^−ΔΔCt^ method [24]. Sequences of specific primers used in this study were: ARAP1-AS1: F: 5′-CTCAGCCCTGTAGAAGCTC-3′, R: 5′-CTGTAGAGGAGCACTCAGC-3′; ARAP1: F: 5′-TCTGTTTGTGCAGCATGAC-3′, R: 5′-CAGAAGAGACAGAGGGTCC-3′; EZH2: F: 5′- AGAATGGAAACAGCGAAGGA-3′, R: 5′-CACCGAACACTCCCTAGTC-3′; GAPDH: F: 5′- TCATTTCCTGGTATGACAACGA-3′, R: 5′-GTCTTACTCCTTGGAGGCC −3′.

### 2.5. Cell proliferation assays

As previously described, 3-(4,5-Dimethylthiazol-2-yl)-2,5-diphenyltetrazolium bromide (MTT; Beyotime, Shanghai, China) was applied to measure LUAD cell viability [25]. LUAD cells were placed in 96-well plates. At 0, 24, 48 and 72 h after transfection, 20 μL of MTT was added. After another 4 h of incubation, 150 μL dimethyl sulfoxide (Sigma-Aldrich, St. Louis, MO, USA) was added and the absorbance at 490 nm was monitored by Microplate Reader (Bio‐Rad, Hercules, CA, USA). For the colony formation assay, LUAD cells were seeded in 6-well plates (1 × 10^3^ cells/well) and maintained in media supplemented with 10% FBS for 2 weeks. The media were replaced every 3 days. After 2 weeks, the colonies were stained with 0.1% crystal violet (Beyotime) after treatment with methanol. Finally, the colonies were observed and counted using a microscope (Olympus Corporation, Center Valley, PA, USA).

### 2.6. Transwell invasion assay

The invasion of transfected A549 and H1975 cells was detected using Matrigel (BD Biosciences, Franklin Lakes, NJ, USA) coated 24-well invasion chambers (8 µM pore size; Corning, Beijing, China) [26]. The upper chamber contained serum-free RPMI 1640 medium and 1 × 10^5^ A549 and H1975 cells, and the lower chamber was filled with full RPMI 1640 medium containing 10% FBS (Gibco). Afterward, the chambers were maintained for 48 h at 37°C in 5% CO_2_. Then, the cells that were attached to the lower surface of the filter membrane were removed by a cotton swab, and the invaded cells were fixed and stained with crystal violet for cell counting. The digital photographs were seized by an Olympus microscope (CX41) at five non-overlapped fields that were selected arbitrarily. Finally, the average number of the invaded LUAD cells was calculated.

### 2.7. Wound healing assay

The migration of transfected LUAD cells was measured through wound healing assay [27]. Cells were seeded into 24‐well plates and incubated for 24 h until 80%-90% confluence. Then, wounds were formed by a 200 μL plastic sterile micropipette tip. The wound width was imaged at 0 and 24 h after wounding with a microscope (Olympus).

### 2.8. Subcellular fractionation assay

Subcellular fraction assay was performed to determine the cellular localization of ARAP1-AS1 in LUAD. Cytoplasmic, nuclear and total RNA was isolated using a PARIS™ kit (Thermo Fisher Scientific, Madison, WI, USA) following the manufacturer’s protocols [28]. RNA from the isolated cytoplasmic and nuclear fractions was reverse transcribed and used for PCR after purification and DNase I treatment. GAPDH served as the endogenous control for the cytoplasm, and U6 for the nucleus.

### 2.9. RNA immunoprecipitation (RIP) assay

RIP assay was conducted using the EZ-Magna RIP kit (Millipore, Billerica, MA, USA) [29]. LUAD cells were lysed in lysis buffer containing RNase inhibitor and protease inhibitor cocktail. A total of 100 μl of whole cell extract was incubated with RIP buffer containing magnetic beads conjugated with anti-IgG and anti-H3K27me3 at 4°C for 6 h. Finally, the coprecipitated RNA was eluted from the beads and analyzed by RT-qPCR.

### 2.10. Western blotting

Radio-immunoprecipitation assay lysis buffer (Beyotime) was used to extract proteins from transfected LUAD cells. Following quantification using a bicinchoninic acid protein assay kit (Beyotime), 10–20 μg protein was transferred to polyvinylidene fluoride membranes (Millipore) for 2 h after being separated by 10% sodium dodecyl sulfate polyacrylamide gel electrophoresis. The membranes were incubated with the primary antibodies against EZH2 (ab191250, 1:1000, Abcam, Cambridge, UK) and ARAP1 (ab99382, 1:2000, Abcam) overnight at 4°C and a secondary antibody against rabbit IgG (ab288151, 1:2000, Abcam) for 1 h at room temperature. The blots were developed using an enhanced chemiluminescence reagent (Millipore) [30].

### 2.11. Luciferase reporter assay

The effects of ARAP1-AS1 or EZH2 on ARAP1 promoter in LUAD cells were detected by luciferase reporter assay. The luciferase reporter plasmids containing ARAP1 promoter were transfected with EZH2-shRNA and EZH2-vector into A549 and H1975 cells using Lipofectamine 2000 (Invitrogen). The ratio of firefly to renilla luciferase activity was detected as the luciferase activity using the dual-luciferase reporter assay system (Promega, Madison, WI, USA) 48 h later [31].

### 2.12. Statistical analysis

SPSS 19.0 software (IBM Corporation, Armonk, NY, USA) was used to analyze all experimental data. Each experiment was repeated in triplicate, and the data are shown as the mean+ standard deviation. Student’s *t*-test was applied for two-group comparison. One-way analysis of variance (ANOVA) followed by Tukey’s *post hoc* test was applied for multiple-group comparison. A value of *p < 0.05* deemed to be statistically significant.

## 3. Results

This study aimed to explore the effects of ARAP1-AS1 on the proliferation, migration and invasion of LUAD cells and the molecular mechanism involved. Through functional experiments, it was demonstrated that ARAP1-AS1 promotes the proliferation, migration and invasion of LUAD cells by silencing ARAP1 through recruiting EZH2.

### ARAP1-AS1 knockdown suppresses LUAD cell growth and metastasis

**Figure 1.**
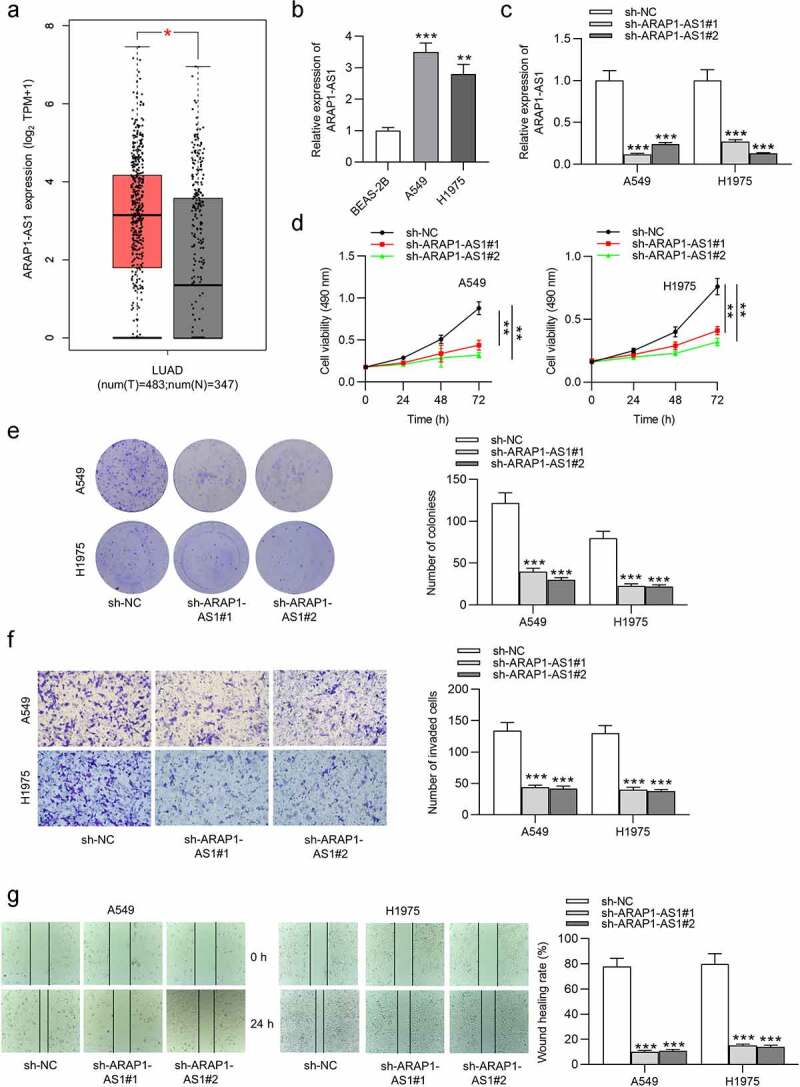
ARAP1-AS1 knockdown suppresses LUAD cell growth and metastasis. (a) GEPIA website revealed ARAP1-AS1 expression in normal tissues (n = 347) and LUAD tissues (n = 483). (b) ARAP1-AS1 expression in LUAD cell lines (A549 and H1975) and a normal lung epithelial cell line (BEAS-2B) were evaluated by RT-qPCR. (c) The interfering efficiency of ARAP1-AS1 in LUAD cells was subjected to RT-qPCR. (d-e) MTT and colony formation assays were performed to detect LUAD cell proliferation after ARAP1-AS1 downregulation. (f-g) LUAD cell invasion and migration after ARAP1-AS1 knockdown were respectively measured by Transwell invasion and wound healing assays. (*) p < 0.05, (**) p < 0.01, (***) p < 0.001.

GEPIA website revealed that ARAP1-AS1 was upregulated in LUAD tissues compared to normal tissues (Figure 1a). Compared with control BEAS-2B cells, A549 and H1975 cells expressed higher ARAP1-AS1 levels (Figure 1b). To evaluate the detailed function of ARAP1-AS1, we conducted several loss-of-function experiments. Specific shRNAs targeting ARAP1-AS1 (sh-ARAP1-AS1#1/2) were employed to interfere ARAP1-AS1 expression in LUAD cells. RT-qPCR indicated that both A549 and H1975 cells showed significantly decreased ARAP1-AS1 expression after ARAP1-AS1 silencing (Figure 1c). ARAP1-AS1 knockdown inhibited the viability of LUAD cells, as shown by MTT assays (Figure 1d). Furthermore, LUAD cell proliferation was also suppressed after downregulating ARAP1-AS1 (Figure 1e). Transwell assays revealed that ARAP1-AS1 downregulation suppressed LUAD cell invasion (Figure 1f). Additionally, wound healing assays showed that ARAP1-AS1 downregulation inhibited LUAD cell migration compared to sh-NC group (Figure 1g).

### ARAP1-AS1 overexpression facilitates LUAD cell growth and metastasis

**Figure 2.**
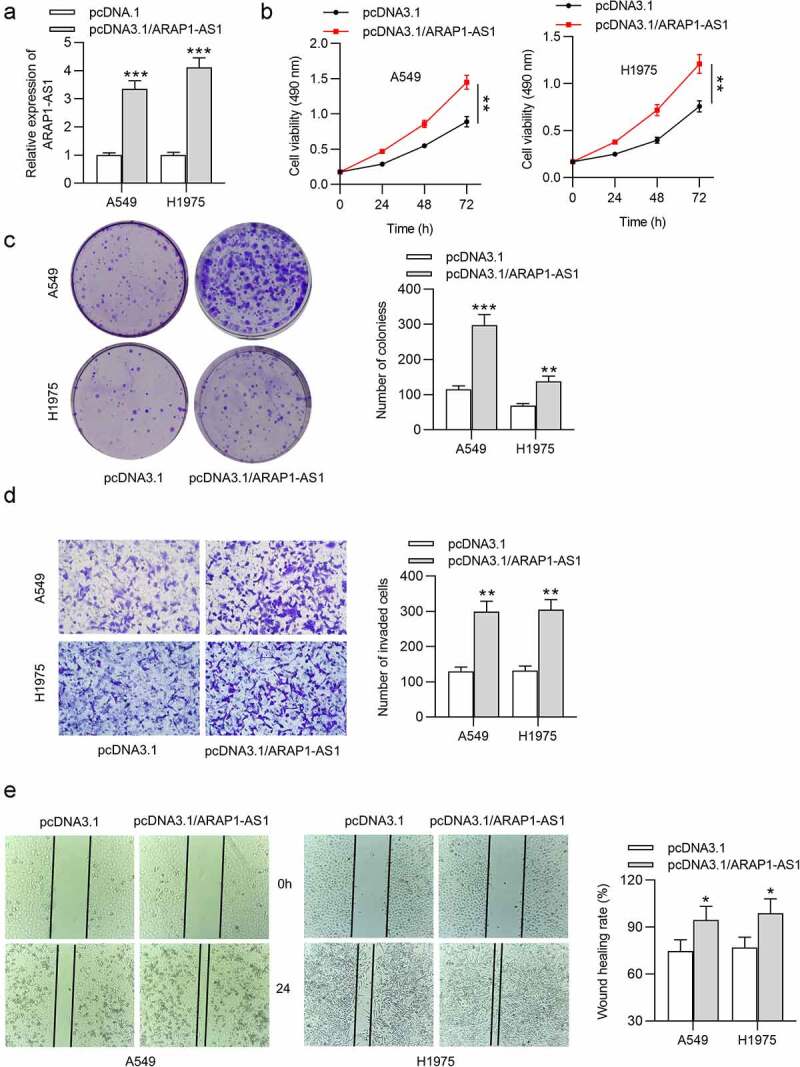
ARAP1-AS1 overexpression facilitates LUAD cell growth and metastasis. (a) The overexpression efficiency of ARAP1-AS1 in LUAD cells was detected by RT-qPCR. (b-c) MTT and colony formation assays were performed to measure LUAD cell proliferation after ARAP1-AS1 overexpression. (d-e) LUAD cell invasion and migration after overexpressing ARAP1-AS1 were respectively examined by Transwell invasion assay and wound healing assay. (*) p < 0.05, (**) p < 0.01, (***) p < 0.001.

Next, we also conducted gain-of-function assays to figure out the effects of ARAP1-AS1 overexpression on LUAD cell phenotypes. As shown by RT-qPCR, the expression of ARAP1-AS1 was elevated in LUAD cells after overexpressing ARAP1-AS1 (Figure 2a). Through functional assays, we discovered that ARAP1-AS1 overexpression significantly promoted LUAD cell proliferation, migration and invasion (Figure 2b-e).

### ARAP1-AS1 negatively modulates ARAP1 expression

**Figure 3.**
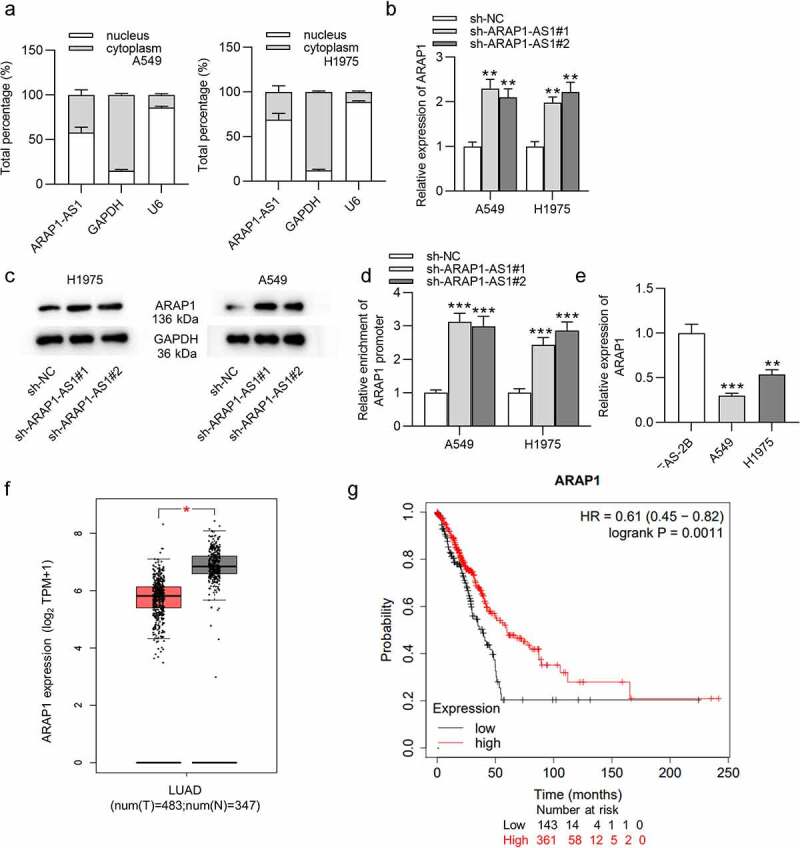
ARAP1-AS1 negatively modulates ARAP1 expression. (a) Subcellular fraction assay was performed for determining the cellular localization of ARAP1-AS1 in LUAD, with U6 and GAPDH as internal controls. (b-c) The influencing of ARAP1-AS1 silencing on mRNA and protein levels of its sense RNA ARAP1 were evaluated by RT-qPCR and western blotting. (d) The luciferase activity of plasmids containing ARAP1 promoter in LUAD cells after ARAP1-AS1 downregulation was evaluated by luciferase reporter assay. (e) ARAP1 expression in LUAD cells versus normal cells was assessed by RT-qPCR. (f) ARAP1 expression in normal tissues (n = 347) and LUAD tissues (n = 483) was shown at GEPIA website. (g) The correlation between ARAP1 expression level and poor prognosis was predicted according to Kaplan-Meier Plotter website. (*) p < 0.05, (**) p < 0.01, (***) p < 0.001.

The cellular localization of ARAP1-AS1 in LUAD was determined by subcellular fraction assay, which showed that ARAP1-AS1 was mainly distributed in the nucleus of LUAD cells (Figure 3a). Then, we detected the influence of ARAP1-AS1 knockdown on its sense mRNA ARAP1. RT-qPCR demonstrated that ARAP1 expression was significantly increased after downregulation of ARAP1-AS1 (Figure 3b). The protein level of ARAP1 was also upregulated by ARAP1-AS1 knockdown according to western blotting results (Figure 3c). Furthermore, luciferase activity of plasmids containing ARAP1 promoter was enhanced in LUAD cells after downregulating ARAP1-AS1 (Figure 3d). ARAP1 expression was found to be reduced in LUAD cells compared with normal cells by using RT-qPCR (Figure 3e). According to GEPIA website, ARAP1 was also downregulated in LUAD tissues relative to normal tissues (Figure 3f). Additionally, LUAD patients with high expression ARAP1 were predicted to have better overall survival than those with low expression ARAP1 according to Kaplan-Meier Plotter website (Figure 3g). In summary, ARAP1-AS1 knockdown positively regulated ARAP1, which was decreased in LUAD tissues and cells.

### ARAP1-AS1 recruits EZH2 to regulate ARAP1 expression

**Figure 4.**
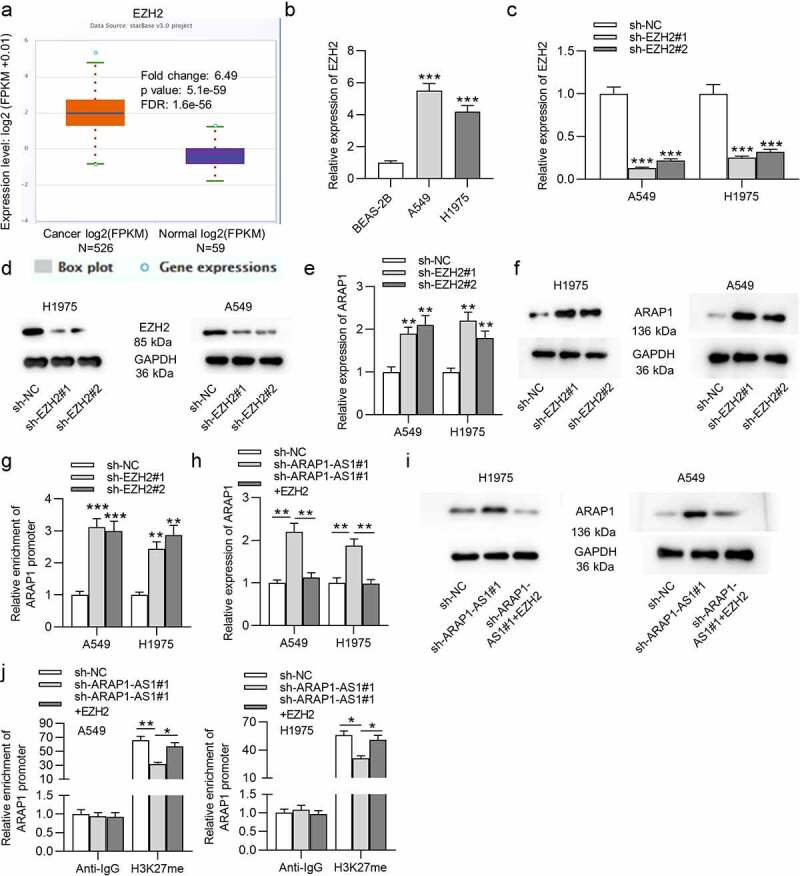
ARAP1-AS1 recruits EZH2 to regulate ARAP1 expression. (a) EZH2 expression in normal tissues (n = 59) and LUAD tissues (n = 526) was predicted at starBase website. (b) RT-qPCR was employed to detect EZH2 expression in LUAD cells versus normal cells. (c-d) The knockdown efficiency of EZH2 in LUAD cells was subjected to RT-qPCR and western blotting. (e-f) RT-qPCR and western blotting were carried out to detect the effects of EZH2 knockdown on ARAP1 expression and protein level. (g) A luciferase reporter assay was performed to measure the luciferase activity of plasmids containing ARAP1 promoter in LUAD cells after EZH2 knockdown. (h-i) The influence of EZH2 overexpression on ARAP1 expression in sh-ARAP1-AS1 transfected LUAD cells. (j) RIP assay was applied to evaluate the enrichment of ARAP1 promoter in H3K27 group in LUAD cells transfected with sh-ARAP1-AS1#1 and pcDNA3.1/EZH2. (*) p < 0.05, (**) p < 0.01, (***) p < 0.001.

According to starBase website, EZH2 expression is higher in LUAD tissues than in normal tissues (Figure 4a). EZH2 expression was also found to be elevated in LUAD cells compared to normal cells (Figure 4b). Then, specific shRNAs targeting EZH2 (sh-EZH2#1/2) were used to knockdown EZH2 in LUAD cells. The interfering efficiency was detected by RT-qPCR (Figure 4c). The protein level of EZH2 was also reduced in LUAD cells transfected with sh-EZH2#1/2, as shown by western blotting (Figure 4d). Next, we analyzed the effects of EZH2 knockdown on ARAP1. We discovered that the expression and protein level of ARAP1 in LUAD cells were both elevated after downregulating EZH2 (Figure 4e-f). In addition, luciferase activity of plasmids containing ARAP1 promoter was also discovered to be higher in LUAD cells after EZH2 downregulation (Figure 4g). Nevertheless, the cotransfection of sh-ARAP1-AS1#1 and pcDNA3.1/EZH2 abolished the promotive effects of ARAP1-AS1 knockdown on ARAP1 expression and protein levels (Figure 4h-i). Additionally, RIP assay revealed that ARAP1-AS1 regulated ARAP1 expression through recruiting EZH2 to its promoter region and elevating the H3K27me enrichment of its promoter (Figure 4j). Taken together, ARAP1-AS1 recruited EZH2 to regulate ARAP1 expression.

### ARAP1 knockdown reverses the inhibition of ARAP1-AS1 on LUAD cell malignant behaviors

**Figure 5.**
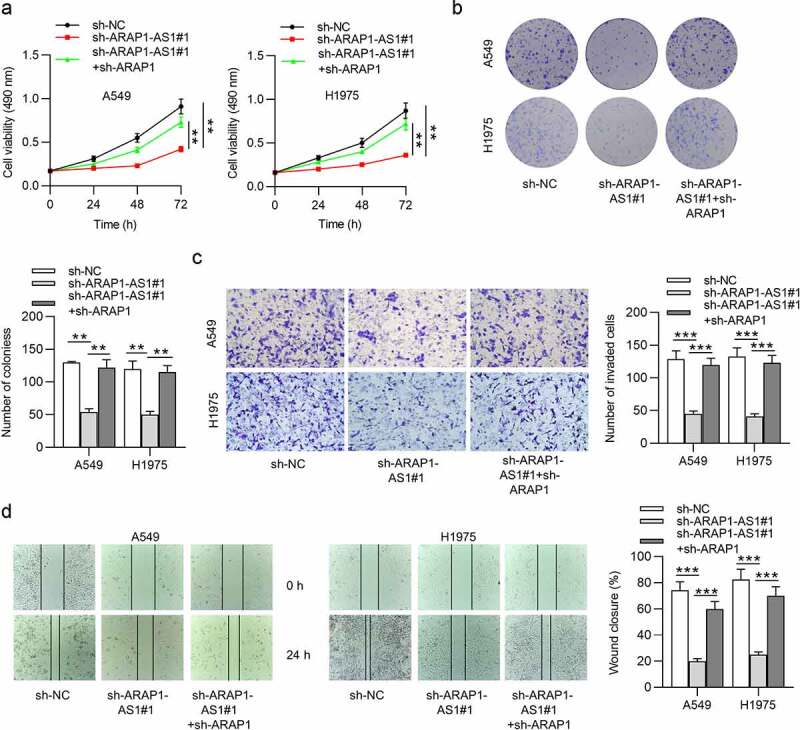
ARAP1 knockdown reverses the inhibition of ARAP1-AS1 on LUAD cell malignant behaviors. (a-b) MTT and colony formation assays were conducted to measure LUAD cell proliferation in sh-NC, sh- ARAP1-AS1#1, sh-ARAP1-AS1#1 + sh-ARAP1 groups. (c-d) Transwell invasion and wound healing assays were performed to evaluate LUAD cell invasion and migration in the above three groups. (*) p < 0.05, (**) p < 0.01, (***) p < 0.001.

To investigate whether ARAP1 is a functional target of ARAP1-AS1, LUAD cells were cotransfected with sh-ARAP1-AS1+ sh-ARAP1. The results demonstrated that silencing of ARAP1-AS1 suppressed LUAD cell proliferation, migration, and invasion. Nevertheless, ARAP1 knockdown abolished the inhibition of ARAP1-AS1 knockdown on the malignant behaviors of A549 and H1975 cells (Figure 5a-d).

## 4. Discussion

LUAD has increased incidence over the past years and comprises almost 50% of lung cancer mortality [32]. Accumulating studies have emphasized the significance of lncRNAs in the pathogenesis of many cancers, including LUAD [33–35]. Herein, we explored the detailed function of lncRNA ARAP1-AS1 in LUAD. The findings indicated that ARAP1-AS1 served as an oncogene in LUAD, which epigenetically silenced ARAP1 by recruiting EZH2 to promote LUAD cell growth and metastasis.

The detailed role and regulatory mechanism of ARAP1-AS1 as a tumor promoter was reported previously. In cervical cancer, ARAP1-AS1 is enhanced in tumor samples, and ARAP1-AS1 overexpression enhances the viability, migration, and invasion of tumor cells via downregulating microRNA-149-3p and elevating the expression of POU class 2 homeobox 2 [36]. ARAP1-AS1 is overexpressed in breast cancer cells and ARAP1-AS1 silencing results in inhibited proliferation, blocked migration, and strengthened apoptosis of breast cancer cells via the microRNA-2110/histone deacetylase 2/perilipin 1 axis [37]. ARAP1-AS1, which shows high expression in colorectal cancer cells and tissues, can be enhanced by YY1 transcription factor to facilitate tumor cell migration, invasion, and epithelial-mesenchymal transition [38]. Importantly, ARAP1-AS1 was previously demonstrated to be highly expressed in lung cancer tissues and cells, and ARAP1-AS1 knockdown markedly inhibited the proliferation of lung cancer cells and induced cell cycle arrest by decreasing the expression of cell cycle-related protein cyclin D1 [39]. However, the influence and underlying molecular mechanism of ARAP1-AS1 on lung cancer cell migration and invasion were not elucidated. In our study, we discovered that ARAP1-AS1 was upregulated in LUAD cells and tissues. ARAP1-AS1 downregulation repressed while ARAP1-AS1 overexpression promoted LUAD cell proliferation. These results are in consistent with those in the previous study. In addition, our study also investigated the influence of ARAP1-AS1 on LUAD cell migration and invasion. LUAD cell migratory and invasive capabilities were remarkably enhanced after overexpressing ARAP1-AS1, but were attenuated after downregulating ARAP1-AS1. Therefore, our study innovatively discovered the promotion of ARAP1-AS1 on LUAD cell metastasis.

ARAP1, a downstream gene of ARAP1-AS1, is a protein with Rho GAP domains an Arf guanosine triphosphatase-activating protein (GAP) [40]. ARAP1 plays a significant role in the internalization and recycling of epidermal growth factor receptor (EGFR) [41]. EGFR is identified as the first one to be associated with cancer development among all receptor tyrosine kinases [42]. The overactivation and overexpression of EGFR are related to cancer metastasis, drug resistance, poor prognosis, and lower survival rate [42]. Our finding revealed that ARAP1 is downregulated in LUAD cells and tissues, and its downregulation predicts poor prognosis of LUAD patients. ARAP1-AS1 was shown to exert an inhibitory effect on ARAP1 expression.

EZH2, as an enzymatic catalytic subunit of polycomb repressive complex 2 (PRC2), can alter the expression of downstream target genes by trimethylation of Lys-27 in histone 3 (H3K27me3) or other ways [43]. It has been widely reported that EZH2 can be recruited by lncRNAs to regulate the development of lung cancer. For example, lncRNA prostate cancer associated transcript 6 promotes non-small-cell lung cancer cell growth, migration and invasion by repressing the expression of large tumor suppressor kinase 2 via binding with the epigenetic repressor EZH2 [44]. LncRNA X inactivate-specific transcript interacts with EZH2 to suppress transcription of its potential target kruppel like factor 2, thereby enhancing non-small-cell lung cancer cell proliferation, migration and invasion [45]. LncRNA 00152 was demonstrated to repress interleukin 24 expression via interacting with EZH2, thus facilitating LUAD cell proliferation and suppressing cell apoptosis [46]. In the previous study, ARAP1-AS1 was discovered to recruit EZH2 to regulate the expression of dual specificity phosphatase 5, a downstream gene of ARAP1-AS1, therefore affecting the development of cervical cancer [47]. Therefore, we inferred whether ARAP1-AS1 recruited EZH2 to regulate the expression of ARAP1 in LUAD. The findings in this study revealed that ARAP1 expression was increased by ARAP1-AS1 knockdown and further decreased by EZH2 overexpression. Thus, we concluded that ARAP1-AS1 suppressed ARAP1 expression by recruiting EZH2. For the first time, our study discovered that ARAP1-AS1 exert its oncogene role in LUAD by repressing ARAP1 expression via recruiting EZH2.

To be honest, there exist some limitations in our study. First, the role of ARAP1-AS1 in LUAD was just confirmed by *in vitro* assays, *in vivo* studies are required to further confirm whether ARAP1-AS1 is an oncogenic lncRNA in LUAD. Second, we have clarified before that the TGF-β/Smad3 pathway, which is associated with the development of some human cancers, can be activated via persistent transactivation of EGFR after ARAP1 regulation. Therefore, the potential signaling pathways related to LUAD tumorigenesis or progression needs further investigation.

## 5. Conclusion

In summary, our study discovered that lncRNA ARAP1-AS1 recruited E2Z1 to epigenetically inhibit ARAP1 expression, thereby promoting the growth and metastasis of LUAD cells. Although there are some limitations, the study might provide a novel therapeutic target and shed a promising light for the future treatment of LUAD.
